# From the tyrosine hydroxylase hypothesis of Parkinson’s disease to modern strategies: a short historical overview

**DOI:** 10.1007/s00702-022-02488-3

**Published:** 2022-04-23

**Authors:** Wolf-Dieter Rausch, Feixue Wang, Khaled Radad

**Affiliations:** 1 Institute of Medical Biochemistry, University of Veterinary Medicine Vienna, Vienna, Austria; 2 Department of Traditional Chinese Medicine, Xuanwu Hospital, Capital Medical University, Beijing, China; 3 Department of Pathology, Faculty of Veterinary Medicine, Assiut University, Assiut, Egypt

**Keywords:** Parkinson’s disease, Tyrosine hydroxylase, Neurodegeneration, Neuroprotection

## Abstract

A time span of 60 years covers the detection of catecholamines in the brain, their function in movement and correlation to Parkinson’s disease (PD). The clinical findings that orally given l-DOPA can alleviate or even prevent akinesia gave great hope for the treatment of PD. Attention focused on the role of tyrosine hydroxylase (TH) as the rate-limiting enzyme in the formation of catecholamines. It became evident that the enzyme driven formation is lowered in PD. Such results could only be obtained from studying human brain samples demonstrating the necessity for human brain banks. Originally, a TH enzyme deficiency was suspected in PD. Studies were conducted on the enzyme properties: its induction and turnover, the complex regulation starting with cofactor requirements as tetrahydrobiopterin and ferrous iron, and the necessity for phosphorylation for activity as well as inhibition by toxins or regulatory feedback inhibition by catecholamines. In the course of time, it became evident that neurodegeneration and cell death of dopaminergic neurons is the actual pathological process and the decrease of TH a cophenomenon. Nevertheless, TH immunochemistry has ever since been a valuable tool to study neuronal pathways, neurodegeneration in various animal models of neurotoxicity and cell cultures, which have been used as well to test potential neuroprotective strategies.

## Introduction

Since the early description of PD by James Parkinson in 1817, scientists directed their efforts to find therapies that could improve the symptoms of the disease, rigidity, tremor and akinesia. As the result, they succeeded to discover and introduce levodopa into the clinic, the most effective symptomatic treatment of PD (Birkmayer and Hornykiewicz 1961; Hornykiewicz 2010). Bernheimer et al. (1973) described neuronal loss of nerve cells in the substantia nigra (SN) with a reduction of dopamine. Demonstration and mapping of nigrostriatal neurons had been performed already earlier by Anden et al. (1964). The cellular localization of TH was proven by Pickel et al. (1975). Labeled cell bodies and their processes were easily distinguished from unstained neuronal elements in the neuropil. TH presence was only seen in the neuronal cytoplasm. Its distribution in processes was different from that in cell soma. In longitudinal sections of axons and dendrites, the peroxidase reaction product appeared as fiber-like strands (Pickel et al. 1975). TH was immunohistochemically localized by the peroxidase–antiperoxidase method in rat to chromaffin cells of the rat adrenal medulla, large neurons and small darkly staining cells of the superior cervical ganglia and noradrenergic and dopaminergic neurons in brain. Thus, experimentally dopaminergic projection areas in mouse and rat brains can be localized as well as dissected. Embryonic cell cultures of midbrain preparations represent a tool for exposing dopaminergic cells in a mixed culture with other neurons and glial elements to toxic compounds as well as neuroprotective agents (Radad et al. 2006).

## TH characteristics and determination

TH has first been described in a detailed paper by Nagatsu et al. (1964). In detail, the paper describes the isolation of enzyme, necessity for its cofactors tetrahydrobiopterin and ferrous iron and its presence in different tissues as adrenal medulla, spleen, and various brain areas of laboratory animals. The intracellular localization could be studied because there is a soluble form of TH and enzyme attached to particles.

This paper and many more to come used enzyme determination steps, which were based on radioactive tyrosine to determine labeled l-Dopa (Levine et al. 1984). Other assays using tyrosine and determining l-Dopa formed by extraction steps over alumina columns and displacement by acid and consecutive determination of l-Dopa by high-performance liquid chromatography systems with electrochemical detection were developed (Naoi et al. 1988).

## TH activity in PD

TH activity was assayed radioenzymatically in various regions of post-mortem brains of human individuals without neurologic disorders (controls), with PD, senile dementia, hypertensive encephalopathy, hepatic and diabetic coma, liver cirrhosis without coma, and hepatic coma treated with parenteral administration of l-valine. In addition, TH activity of the post-mortem adrenal medulla was assayed in controls, PD, senile dementia and hypertensive encephalopathy. In PD, activity of TH was significantly decreased in the nigrostriatal system, and less severe in other brainstem areas, while the raphe-reticular formation and limbic system showed normal values. In addition, there was significant decrease in the TH activity of the adrenal medulla, suggesting that PD is a generalized disorder not limited to distinct CNS areas (Riederer et al. 1978).

Such studies proved that TH is a relevant enzyme in PD and related disorders (Nagatsu et al. 2019) as well demonstrate that human brain biochemistry has manifold caveats which should be considered. Ethical and religious objections are just one issue. Age, ongoing neurodegenerative processes as well as long-term medication are relevant. In addition, premortem comatose states, post-mortem times in between death and dissection and storage conditions have influence on obtained values.

Nevertheless, findings of decreased TH activity in PD raised the hope that PD was partially a lack or defect of TH and stimulation of TH could compensate functional deficits (Tolleson and Claassen 2012). Detailed studies exist on the activation/inactivation of TH by phosphorylation in animal tissues (Shehadeh et al. 2019). Using the same techniques, stimulation could not be detected in cAMP, ATP, protein kinase and Ca-Calmodulin systems, suggesting that these systems are not responsive in human brain tissue any more (Rausch et al. 1988).

Interestingly, however, remaining TH enzyme in PD brain has compensatory ability. Mogi et al. (1988) could show that TH “homospecific activity” (activity per enzyme protein) was significantly increased in the parkinsonian brains whereas TH protein content and TH activity were decreased compared to controls. This increase suggests that activity changes occur in the TH molecule. An actual mechanism is not known so far, but a connection to iron metabolism seems possible.

## The role of iron for TH

Stimulation of TH by ferrous iron has already been found in the early paper on characterization of this enzyme by Nagatsu et al. (1964). Iron-dependent stimulation was shown in these initial studies on the kinetics of the purified enzyme. Haavik et al. (1991) described that human TH exists as four different isozymes (TH1–TH4), generated by alternative splicing of pre-mRNA. When expressed in *Escherichia coli*, the purified isozymes revealed high catalytic activity when reconstituted with Fe(II) and stability at neutral pH. The isozymes contained 0.04–0.1 atom iron and 0.02–0.06 atom zinc/enzyme subunit. All isozymes were rapidly activated (13–40-fold) by incubation with Fe(II) salts and inhibited by other divalent metal ions, e.g., Zn(II), Co(II) and Ni(II). They all bind stoichiometric amounts of Fe(II) and Zn(II) with high affinity (*K*_d_ = 0.2–3 μM at pH 5.4–6.5). Similar time courses were observed for binding of Fe(II) and enzyme activation. In the absence of any free Fe(II) or Zn(II), the metal ions dissociated from the reconstituted isozymes. The process was favored by acidic pH, as well as by the presence of metal chelators and dithiothreitol.

TH in human control tissues was stimulated by 1 mM iron (II) 13-fold. Although the activity of TH in striata of PD was 60% of controls, stimulation with 1 mM iron (II) reached an 11-fold increase of TH activity. This finding was similar to that of controls as was the Km-value of TH in controls and PD (Rausch et al. 1988; Riederer et al. 1988). Interestingly, human TH activities were about 100-fold lower than rat tissue, however, the ferrous iron stimulation in animal brain was much less pronounced. Soluble TH interacts with alpha-synuclein (ɑ-syn) and an increase of ɑ-syn leads to reduction of TH activity (Kaushik et al. 2007; Olivares et al. 2009). Therefore, it is hypothesized that iron-induced enhancement of TH activity by reducing ɑ-syn activity increases dopamine concentration. Furthermore, reaction of dopamine with iron causes ɑ-syn aggregation (Sian-Hulsmann et al. 2015). In conclusion, “iron” is a major player in the pathology of PD (Berg et al. 2001; Sofic et al. 1988). Chelation of excess iron at the site of the substantia nigra, where a dysfunction of the BBB is suggested, with peripherally acting iron chelators is suggested to contribute to the portfolio and therapeutic armamentarium of anti-Parkinson medications (Riederer et al. 2021).

## Neuronal death of dopaminergic neurons

Neuronal cell death is a phenomenon accompanying age. Adult neuronal cells do not divide anymore and are thus exposed for life to the milieu inside the brain, which will change over time. In case of TH-positive neurons, remarkable length of axons bridges the cell bodies in the substantia nigra to the target regions in caudate and putamen. Causes of death thus may even be mechanic stress during osmotic changes or shrinkage in the brain. Dopaminergic neurons as well depend on their oxygen supply. A considerable part of PD cases according to Udagedara et al. (2019) is considered to be caused by cerebrovascular changes. A wide body of studies claimed environmental toxins such as herbicides, pesticides and natural toxins to be potentially responsible for neuronal cell death (Bové et al. 2005; Salama and Arias-Carrion 2011).

Studies on a drug related to heroin, 1-Methyl-4-phenyl-1,2,3,6-tetrahydropyridine (MPTP), have gained wide attention. MPTP is a neurotoxin, which causes symptoms of PD in humans and animals. Metabolized by monoamine oxidase the product 1-Methyl-4-phenylpyridinium (MPP^+^) inhibits the mitochondrial respiratory chain and destroys dopaminergic cells in the substantia nigra (Liu et al. 2020; Zhu et al. 2019).

The etiology of PD remains unknown. Genetic, environmental risk factors and their interaction play a major role for PD. Although 90% of PD cases are sporadic, several genes exist which are directly related to inherited cases of PD (Blauwendraat et al. 2020; Puschmann 2017). Mutations in the α-syn-gene and the leucine-rich repeat kinase LRRK2 (PARK8 gene) cause autosomal dominant PD, while mutations in Parkin (PARK2 gene), PINK1 (PARK6 gene) or DJ-1 (PARK7 gene) cause autosomal recessive PD (Haugarvoll and Wszolek 2006).

To observe the death of dopamine neurons in the human brain as a daily phenomenon seems hardly possible. In cell culture, the disappearance of cells can be documented by shortening of axonal and dendritic processes, lost cells and remaining apoptotic patches (Lotharius et al. 1999). In this context, Callizot et al. (2019) studied the link of mitochondrial dysfunction and aggregation of α-syn on mechanisms of dopaminergic neuronal death. In rat primary cultures of mesencephalic neurons, mitochondrial impairment was induced using “dopamine-toxins” (MPP^+^, 6-hydroxydopamine (6-OHDA) or rotenone). Cell death originates through necrosis, apoptosis and necroptosis: 6-OHDA activates caspase-dependent cell death, MPP^+^ stimulates caspase-independent cell death and rotenone activates both pathways (Callizot et al. 2019).

Excitotoxic death by glutamate is as well discussed for midbrain dopaminergic neurons. Timely clearance of glutamate from synaptic clefts is necessary to prevent high levels of extracellular glutamate resulting in excitotoxicity and neuroinflammation (Wang et al. 2020).

## Neuromelanin functions

PD is accompanied by a loss of pigmented neurons in the SN. Neuromelanin (NM) in the presynaptic terminal of dopaminergic neurons has emerged as a primary player in the etiology of neurodegenerative disorders including PD. NM appears to confer susceptibility to chemical toxicity by providing a large sink for iron (Haining and Achat-Mendes 2017). Progressive accumulation of NM with age along with a decrease in dopamine synthetic pathways poses the immediate question whether NM is capable of binding dopamine, the primary functional monoamine utilized (Latif et al. 2021). Such a memory function as a release on need is being proposed. In detailed morphometric studies on brains 4 years post-diagnosis of PD, there was a loss of 50 to 90% of TH-positive neurons. Interestingly, more melanin-containing neurons than TH-positive neurons remained (Kordower et al. 2013). Experimentally, at least synthetic neuromelanin appears toxic to dopaminergic cells in culture (Nguyen et al. 2002). Clinically such findings are supported as neuromelanin sensitive MRI showed a different distribution of melanin and TH-positive cells (Martin-Bastida et al. 2019).

## A role of α-syn and Lewy bodies

α-syn, the main component of Lewy bodies (LBs), regulates the production of dopamine through its interaction with TH. Over-expression of α-syn reduces the levels of TH mRNA and protein in the brain. Feve (2012) extends the findings that not only TH activity but as well TH synthesis and TH mRNA are affected. The opinion is presented that as long as TH is present in remaining dopaminergic neurons; it can still be used as a pharmacological target.

A histological hallmark of PD is the presence of α-syn-positive aggregates called LBs and axonal fibrillar α-syn deposits. LBs’ formation has been considered to be a marker for neuronal degeneration (Dickson 2018; Wakabayashi et al. 2013).

Studies have suggested that oligomers and protofibrils of α-syn are cytotoxic and that LBs may represent a cytoprotective mechanism in PD (Wakabayashi et al. 2007). Nevertheless, to date, it is still impossible to predict how LB formation affects dopaminergic neuron viability in PD patients because of their complex protein composition (Beyer et al. 2009). The mechanism of TH depletion by the ubiquitin–proteasome system contributes to an understanding of the etiology of PD (Kawahata and Fukunaga 2020). A relation of α-syn aggregation with the loss of TH protein in PD is detectable.

## Contribution of autophagy

Dysregulated autophagy, whether excessive or downregulated, has been thought to be associated with neurodegenerative disorders including PD. Accordingly, an early study was carried out to investigate whether 3-methyladenine, an autophagy inhibitor, can modulate the effects of rotenone on dopaminergic neurons in primary mesencephalic cell culture. However, only small effects on the phagocytotic process were found (Radad et al. 2020). Initially looked at as a cell death pathway, it may as well have a survival function through clearing of protein aggregates and damaged cytoplasmic organelles (Radad et al. 2015).

## Effect of neuroinflammation

In PD, clinical and experimental data suggest that neuroinflammatory cytokines released by microglial activation contribute to neuronal death (Hirsch et al. 2012; Campolo et al. 2020). Experimentally, neuroinflammation of dopaminergic neurons can be evoked by lipopolysaccharide (LPS) exposure. In mesencephalic TH-immunoreactive cells, LPS resulted in a 30–50% loss of dendritic processes, changes in the perikarya, cellular atrophy and neuronal cell loss (Lin et al. 2007). Inducible nitric oxide synthase (iNOS) activity was increased dose dependently as well as prostaglandin E2 concentrations. Ginsenosides, as the active compounds responsible for ginseng action, are reported to have antioxidant and anti-inflammatory effects and demonstrated partial neuroprotection.

## Age-related decline of TH in controls and PD

The by far largest number of parkinsonian cases is idiopathic in origin, with no clear causative agent. Cell death over time may follow different courses, either a continuous degenerative course, or an acute event as, e.g., stroke and then continuous decline. The time sequence leading to cell death can be studied when plotting TH activity versus age and comparing it with PD brain values. Using this technique, a linear decline was observed in human brain striatum samples indicating that at the age of 120 years all of us will be parkinsonian (Rausch 1979).

What is necessary in PD treatment, however, is to follow this curve back and look at the early onset of PD. PD is mainly diagnosed at a stage, when a large number of dopaminergic neurons are lost. Therefore, identification of molecular biomarkers for early detection of PD is important. Given that microRNAs are crucial in controlling gene expression, these regulatory microRNAs and their target genes could be used as biomarkers for early diagnosis of PD (Arshad et al. 2017). Given such good diagnostic tools, neuroprotective strategies would be more promising.

## TH as marker for dopaminergic neurons in cell cultures

Cell cultures containing dopaminergic neurons such as primary mesencephalic cell culture and dopaminergic cell lines (e.g., PC12 cell line, SH-SY5Y neuroblastoma cells, N27 rat dopaminergic neural cell line) are relevant in vitro models for PD (Henley et al. 2017). They are also useful to (1) investigate properties and characteristics of dopaminergic neurons (Gaven et al. 2014), (2) reveal the underlying mechanisms mediating degeneration of dopaminergic neurons and (3) test potential neuroprotective compounds (Henley et al. 2017). Scientific community has seen TH as an ideal marker for identification of dopaminergic neurons in different cell cultures. Among different visualization methods, immunohistochemistry using monoclonal TH antibody is an effective and accurate method to mark dopaminergic neurons in different cell cultures (Hédou et al. 2000).

## Neuroprotective strategies

Dopamine receptor agonists have become increasingly popular as antiparkinsonian therapy since the introduction of bromocriptine by Donald Calne and colleagues in 1974 (Foley et al. 2004).

As both l-Dopa and dopamine agonists lack sufficient efficacy and produce pronounced side effects with the progression of the disease (Dhall and Kreitzman 2016), contemporary research aims to develop disease-modifying agents that can slow down or stop the progression of the disease. In this context, many preclinical studies, both in vivo and in vitro, have examined numerous compounds with neuroprotective potential including dopamine receptor agonists, monoamine oxidase-B (MAO-B) inhibitors, anti-inflammatory compounds, herbal formulations and others.

## Dopamine receptor agonists

Both ergoline- (bromocriptine, cabergoline, dihydroergocryptine, lisuride, pergolide) and non-ergoline (pramipexole, ropinirole, rotigotine, piribedil, apomorphine) dopamine agonists were demonstrated to have neuroprotective effects on dopaminergic neurons based largely on experimental evidence in vitro (Iida et al. 1999; Takashima et al. 1999; Gille et al. 2002, 2006; Oster et al. 2014; Reichelt et al. 2016) and in vivo (Muralikrishnan and Mohanakumar 1998; Yoshioka et al. 2002; Scheller et al. 2008). Different mechanisms are involved in neuroprotection by dopamine receptor agonists, most notably, stimulation of dopamine receptors, antioxidant activity, stimulation of P13K/Akt pathway, inactivation of pro-apoptotic factor, de novo protein synthesis, upregulation of metallothionein expression in astrocytes by targeting 5-HT1A receptors (Moldzio et al. 2006; Oster et al. 2014; Reichelt et al. 2016; Isooka et al. 2020).

## MAO-B inhibitors

The use of MAO-B inhibitors for treating PD dates back more than 50 years. It began after the outstanding discovery of a selective inhibitor, selegiline, by Kálmán Magyar (Szöko et al. 2018). As MAO-B is involved mainly in the degradation of dopamine, the inhibitors are used based on their dopamine-sparing activity, i.e., to prolong the action of both endogenously and exogenously derived dopamine (Chen et al. 2007). Studies with selegiline, the gold-standard MAO-B inhibitor, and rasagiline, the second approved MAO-B inhibitor, showed that their neuroprotective and antiapoptotic properties in experimental models involve MAO-B-independent mechanisms. These properties were shown to be connected to the propargylamine structure (Szöko et al. 2018). Moreover, it was shown that neuroprotective properties of selegiline and rasagiline are associated with maintaining mitochondrial function, upregulation of genes coding antioxidant enzymes, antiapoptotic Bcl-2 and pro-survival neurotrophic factors, and oligomerization and aggregation of α-syn in cellular and animal models (Mandel et al. 2003; Naoi et al. 2020; Salamon et al. 2020).

## Non-steroidal anti-inflammatory drugs

A number of studies have concluded that non-steroidal anti-inflammatory drugs (NSAIDs) carry some neuroprotective effects in PD since it had been reported that inflammation is associated with the pathogenesis of the disease (Asanuma et al. 2004). For instance, Casper et al. (2000) and Swiatkiewicz et al. (2013) showed that ibuprofen rescued dopaminergic neurons in a glutamate-treated primary dopaminergic cell culture and in the striatum of MPTP-treated mice. In a clinical epidemiological study, Chen et al. (2003) reported that the regular use of non-aspirin NSAIDs decreased the risk of PD incidence compared to non-regular users. Neuroprotective effect of NSAIDs was reported to account for inhibition of cyclooxygenases and nitric oxide synthesis (Asanuma et al. 2004).

## Herbal formulations

Plant extracts have been used to treat many different illnesses through history (Uddin et al. 2013). More recently, there is growing evidence indicates that constituents of some medicinal plants can be neuroprotective in PD (Fu et al. 2015). In this context, Zhou et al. (2016) showed that ginsenoside Rg1, one of the major ingredients of Panax ginseng, rescued dopaminergic neurons against MPP + and MPTP-induced cell damage in primary mesencephalic cell culture and mice, respectively. Cui et al. (2015) demonstrated that curcumin protected dopaminergic neurons in the SNpc of rotenone-injured rats. According to Lofrumento et al. (2014) and Arbo et al. (2020) resveratrol, a stilbene found in some plants including blueberries, grapes and peanuts, protected dopaminergic neurons in experimental models of PD. Green tea polyphenols were shown to be neuroprotective in many in vitro and in vivo models of PD (Caruana and Vassallo 2015). Moldzio et al. (2010) and Kim et al. (2010) found that green tea polyphenol epigallocatechin-3-gallate (EGCG) protected dopaminergic neurons in primary dopaminergic cell culture and a mouse model of PD stressed with rotenone and MPTP, respectively. Herbal ingredients were reported to have a wide range of underlying neuroprotective mechanisms, most notably antioxidant, anti-inflammatory, dopamine-sparing, antiapoptotic and anti-α-synuclein aggregation actions (Fu et al. 2015; Nebrisi 2021).

## Other neuroprotectants

Many others compounds have been shown to possess possible neuroprotective effects in relation to PD such as some vitamins, creatinine, co-enzyme Q10, caffeine and nicotine. Numerous clinical, animal and cellular studies have shown neuroprotective candidates such as vitamins, particularly A, B, C, D and E, in PD (Zhang et al. 2002). The neuroprotective action of vitamins was reported to be mediated by their antioxidant, anti-inflammatory and gene regulating actions (Zhao et al. 2019). Creatine is an essential compound in maintaining cellular energy and has shown neuroprotection in many acute and chronic experimental models of neurological diseases including PD (Klein and Ferrante 2007). For example, Andres et al. (2005) and Beal (2011) reported that creatine protected dopaminergic neurons against neurotoxic insults induced by serum/glucose deprivation, MPP^+^ and 6-OHDA in primary mesencephalic cell culture and in MPTP-treated mice, respectively. The electron acceptor for complex I and II, and potent antioxidant co-enzyme Q10 was found to save dopaminergic neurons both in vitro and in vivo models of PD. In parallel, Kooncumchoo et al. (2006) protected human dopaminergic SK-N-SH neurons with Q10 against iron-induced apoptotic death. Beal et al. (1998) demonstrated that co-enzyme Q10 reduced loss of dopaminergic axons in the striatum. Caffeine, the most-widely consumed psychoactive substance, was reported to provide neuroprotection in MPTP, 6-OHDA and rotenone-treated animal models (Ren and Chen 2020). Nicotine, the alkaloid derived from tobacco, which was studied more intensely is suggested as a neuroprotective compound in PD based on the negative correlation between tobacco consumption and PD (Barreto et al. 2015). In rotenone-treated primary dopaminergic cell culture, Delijewski et al. (2021) showed that nicotine protected dopaminergic neurons through an antioxidant mechanism.

## Conclusion

This review intended to follow the fate of tyrosine hydroxylase in PD research. Over 60 years of brain research were covered by extracting a selection out of many papers focusing on dopaminergic neurodegeneration. Excellent animal and TH-positive cell culture models have opened a way to test various compounds for toxicity as well as for neuroprotection. Various antiparkinsonian drugs have emerged which supplement the gold-standard l-Dopa such as dopamine agonists and the selective monoamine oxidase inhibitors selegiline and rasagiline. Refinement of medication has resulted in better quality of life for a considerably long time in individual patients. Despite the fact that many models and mechanisms of neurodegeneration have been postulated, the understanding of the actual trigger for the process leading to cell death, however, has not been clarified during the last 60 years. Definitely, early diagnosis before the clinical onset of the disease seems to be an option to start effective neuroprotective treatment in PD. Modern imaging techniques will help to study ongoing neurodegenerative changes. New ways and approaches deserve attention. The role of the gut microbiota and their products for brain activity are still poorly understood. These products may well be capable to provide TH stimulation in the gut and thus more DOPA could reach the brain. Experimentally, dopamine cell cultures will be extended to longer time spans increased sensitivity for biochemical changes can be achieved and studies in a few cells or even single cell approaches will become possible.

Though extensive preclinical studies have been performed by scientists to look for a neuroprotective drug that can slow down or halt the disease progression, neuroprotection in PD is still a major unmet need and only few clinical trials are promising. Therefore, scientists are required to: (1) refine their basic research to determine which agent could be a promising therapeutic drug and (2) change the methodology, develop reliable animal models and establish effective markers to successfully predict the efficacy of putative neuroprotective agents.

## References

[CR1] Anden NE, Carlsson A, Dahlstroehm A (1964). Demonstration and mapping out of nigro-neostriatal dopamine neurons. Life Sci.

[CR2] Andres RH, Huber AW, Schlattner U (2005). Effects of creatine treatment on the survival of dopaminergic neurons in cultured fetal ventral mesencephalic tissue. Neuroscience.

[CR3] Arbo BD, André-Miral C, Nasre-Nasser RG (2020). Resveratrol derivatives as potential treatments for Alzheimer’s and Parkinson’s Disease. Front Aging Neurosci.

[CR4] Arshad AR, Sulaiman SA, Saperi AA (2017). MicroRNAs and target genes as biomarkers for the diagnosis of early onset of Parkinson disease. Front Mol Neurosci.

[CR5] Asanuma M, Miyazaki I, Ogawa N (2004). Neuroprotective effects of nonsteroidal anti-inflammatory drugs on neurodegenerative diseases. Curr Pharm Des.

[CR6] Barreto GE, Iarkov A, Moran VE (2015). Beneficial effects of nicotine, cotinine and its metabolites as potential agents for Parkinson’s disease. Front Aging Neurosci.

[CR7] Beal MF (2011). Neuroprotective effects of creatine. Amino Acids.

[CR8] Beal MF, Matthews RT, Tieleman A (1998). Coenzyme Q10 attenuates the 1-methyl-4-phenyl-1,2,3, tetrahydropyridine (MPTP) induced loss of striatal dopamine and dopaminergic axons in aged mice. Brain Res.

[CR9] Bernheimer H, Birkmayer W, Hornykiewicz O (1973). Brain dopamine and the syndromes of Parkinson and Huntington. J Neurol Sci.

[CR97] Berg  D, Gerlach M, Youdim MB (2001). Brain iron pathways and their relevance to Parkinson's disease. J
Neurochem.

[CR10] Beyer K, Domingo-Sàbat M (2009). Molecular pathology of Lewy body diseases. Int J Mol Sci.

[CR11] Birkmayer W, Hornykiewicz O (1961). The effect of l-3,4- dihydroxyphenylalanine (= DOPA) on akinesia in parkinsonism. Wien Klin Wochenschr.

[CR12] Blauwendraat C, Nalls MA, Singleton AB (2020). The genetic architecture of Parkinson's disease. Lancet Neurol.

[CR13] Bové J, Prou D, Perier C (2005). Toxin-induced models of Parkinson’s disease. Neurotherapeutics.

[CR14] Callizot N, Combes M, Henriques A (2019). Necrosis, apoptosis, necroptosis, three modes of action of dopaminergic neuron neurotoxins. PLoS ONE.

[CR15] Campolo M, Filippone A, Biondo C (2020). TLR7/8 in the pathogenesis of Parkinson’s disease. Int J Mol Sci.

[CR16] Caruana M, Vassallo N (2015). Tea polyphenols in Parkinson's disease. Adv Exp Med Biol.

[CR17] Casper D, Yaparpalvi U, Rempel N (2000). Ibuprofen protects dopaminergic neurons against glutamate toxicity in vitro. Neurosci Lett.

[CR18] Chen H, Zhang SM, Hernán MA (2003). Nonsteroidal anti-inflammatory drugs and the risk of Parkinson disease. Arch Neurol.

[CR19] Chen JJ, Swope DM, Dashtipour K (2007). Comprehensive review of rasagiline, a second-generation monoamine oxidase inhibitor, for the treatment of Parkinson's disease. Clin Ther.

[CR20] Cui Q, Li X, Zhu H (2015). Curcumin ameliorates dopaminergic neuronal oxidative damage via activation of the Akt/Nrf2 pathway. Med Rep.

[CR21] Delijewski M, Radad K, Krewenka C (2021). The reassessed impact of nicotine against neurotoxicity in mesencephalic dopaminergic cell cultures and neuroblastoma N18TG2 cells. Planta Med.

[CR22] Dhall R, Kreitzman DL (2016). Advances in levodopa therapy for Parkinson disease: review of RYTARY (carbidopa and levodopa) clinical efficacy and safety. Neurology.

[CR23] Dickson DW (2018). Neuropathology of Parkinson disease. Parkinsonism Relat Disord.

[CR24] Feve AP (2012). Current status of tyrosine hydroxylase in management of Parkinson's disease. CNS Neurol Disord Drug Targets.

[CR25] Foley P, Gerlach M, Double KL (2004). Dopamine receptor agonists in the therapy of Parkinson’s disease. J Neural Transm.

[CR26] Fu W, Zhuang W, Zhou S (2015). Plant-derived neuroprotective agents in Parkinson’s disease. Am J Transl Res.

[CR27] Gaven F, Marin P, Claeysen S (2014). Primary culture of mouse dopaminergic neurons. J vis.

[CR28] Gille G, Rausch WD, Hung ST (2002). Pergolide protects dopaminergic neurons in primary culture under stress conditions. J Neural Transm.

[CR29] Gille G, Radad K, Reichmann H (2006). Synergistic effect of alpha-dihydroergocryptine and L-dopa or dopamine on dopaminergic neurons in primary culture. J Neural Transm.

[CR30] Haavik J, Le Bourdelles B, Martinez A (1991). Recombinant human tyrosine hydroxylase isozymes. Reconstitution with iron and inhibitory effect of other metal ions. Eur J Biochem.

[CR31] Haining RL, Achat-Mendes C (2017). Neuromelanin, one of the most overlooked molecules in modern medicine, is not a spectator. Neural Regen Res.

[CR32] Haugarvoll K, Wszolek ZK (2006). PARK8 LRRK2 parkinsonism. Curr Neurol Neurosci Rep.

[CR33] Hédou G, Chasserot-Golaz S, Kemmel V, et al (2000) Immunohistochemical studies of the localization of neurons containing the enzyme that synthesizes dopamine, GABA, or γ-hydroxybutyrate in the rat substantia nigra and striatum. J Comp Neur 426:549-560. 10.1002/1096-9861(20001030)426:4<549::aid-cne4>3.0.co;2-y 10.1002/1096-9861(20001030)426:4<549::aid-cne4>3.0.co;2-y 11027398

[CR34] Henley BM, Cohen BN, Kim CH (2017). Reliable identification of living dopaminergic neurons in midbrain cultures using RNA sequencing and TH-promoter-driven eGFP expression. J vis Exp.

[CR35] Hirsch EC, Vyas S, Hunot S (2012). Neuroinflammation in Parkinson's disease. Parkinsonism Relat Disord.

[CR36] Hornykiewicz O (2010). A brief history of levodopa. J Neurol.

[CR37] Iida M, Miyazaki I, Tanaka K (1999). Dopamine D2 receptor-mediated antioxidant and neuroprotective effects of ropinirole, a dopamine agonist. Brain Res.

[CR38] Isooka N, Miyazaki I, Kikuoka R (2020). Dopaminergic neuroprotective effects of rotigotine via 5-HT1A receptors: Possibly involvement of metallothionein expression in astrocytes. Neurochem Int.

[CR39] Kaushik P, Gorin F, Vali S (2007). Dynamics of tyrosine hydroxylase mediated regulation of dopamine synthesis. J Comput Neurosci.

[CR40] Kawahata I, Fukunaga K (2020). Degradation of tyrosine hydroxylase by the ubiquitin-proteasome system in the pathogenesis of Parkinson's disease and Dopa-responsive dystonia. Int J Mol Sci.

[CR41] Kim JS, Kim JM, Jeon BS (2010). Inhibition of inducible nitric oxide synthase expression and cell death by (-)-epigallocatechin-3-gallate, a green tea catechin, in the 1-methyl-4-phenyl-1,2,3,6-tetrahydropyridine mouse model of Parkinson’s disease. J Clin Neurosci.

[CR42] Klein AM, Ferrante RJ (2007). The neuroprotective role of creatine. Subcell Biochem.

[CR43] Kooncumchoo P, Sharma S, Porter J (2006). Coenzyme Q_10_ provides neuroprotection in iron-induced apoptosis in dopaminergic neurons. J Mol Neurosci.

[CR44] Kordower JH, Olanow CW, Dodiya HB (2013). Disease duration and the integrity of the nigrostriatal system in Parkinson's disease. Brain.

[CR45] Latif S, Jahangeer M, Maknoon Razia D (2021). Dopamine in Parkinson's disease. Clin Chim Acta.

[CR46] Levine RA, Pollard HB, Kuhn DM (1984). A rapid and simplified assay method for tyrosine hydroxylase. Anal Biochem.

[CR47] Lin WM, Zhang YM, Moldzio R (2007). Ginsenoside Rd attenuates neuroinflammation of dopaminergic cells in culture. J Neural Transm.

[CR48] Liu WW, Wei SZ, Huang GD (2020). BMAL1 regulation of microglia-mediated neuroinflammation in MPTP-induced Parkinson's disease mouse model. FASEB J.

[CR49] Lofrumento DD, Nicolardi G, Cianciulli A (2014). Neuroprotective effects of resveratrol in an MPTP mouse model of Parkinson's-like disease: possible role of SOCS-1 in reducing pro-inflammatory responses. Innate Immun.

[CR50] Lotharius J, Dugan LL, O'Malley KL (1999). Distinct mechanisms underlie neurotoxin-mediated cell death in cultured dopaminergic neurons. J Neurosci.

[CR51] Mandel S, Grünblatt E, Riederer P (2003). Neuroprotective strategies in Parkinson’s disease: an update on progress. CNS Drugs.

[CR52] Martín-Bastida A, Lao-Kaim NP, Roussakis AA (2019). Relationship between neuromelanin and dopamine terminals within the Parkinson's nigrostriatal system. Brain.

[CR53] Mogi M, Harada M, Kiuchi K (1988). Homospecific activity (activity per enzyme protein) of tyrosine hydroxylase increases in parkinsonian brain. J Neural Transm.

[CR54] Moldzio R, Radad K, Duvigneau JC (2006). Glutamate-induced cell death and formation of radicals can be reduced by lisuride in mesencephalic primary cell culture. J Neural Transm.

[CR55] Moldzio R, Radad K, Krewenka C (2010). Effects of epigallocatechin gallate on rotenone-injured murine brain cultures. J Neural Transm.

[CR56] Muralikrishnan D, Mohanakumar KP (1998). Neuroprotection by bromocriptine against 1-methyl-4-phenyl-1,2,3,6-tetrahydropyridine-induced neurotoxicity in mice. FASEB J.

[CR57] Nagatsu T, Levitt M, Udenfriend S (1964). Tyrosine hydroxylase: the initial step in norepinephrine biosynthesis. J Biol Chem.

[CR58] Nagatsu T, Nakashima A, Ichinose H (2019). Human tyrosine hydroxylase in Parkinson's disease and in related disorders. J Neural Transm.

[CR59] Naoi M, Takahashi T, Nagatsu T (1988). Simple assay procedure for tyrosine hydroxylase activity by high-performance liquid chromatography employing coulometric detection with minimal sample preparation. J Chromatogr.

[CR60] Naoi M, Maruyama W, Shamoto-Nagai M (2020). Rasagiline and selegiline modulate mitochondrial homeostasis, intervene apoptosis system and mitigate α-synuclein cytotoxicity in disease-modifying therapy for Parkinson's disease. J Neural Transm.

[CR61] Nebrisi EE (2021). Neuroprotective activities of curcumin in Parkinson's disease: A review of the literature. Int J Mol Sci.

[CR62] Nguyen A, Gille G, Moldzio R (2002). Synthetic neuromelanin is toxic to dopaminergic cell cultures. J Neural Transm.

[CR63] Olivares D, Huang X, Branden L (2009). Physiological and pathological role of alpha-synuclein in Parkinson's disease through iron mediated oxidative stress; the role of a putative iron-responsive element. Int J Mol Sci.

[CR64] Oster S, Radad K, Scheller D (2014). Rotigotine protects against glutamate toxicity in primary dopaminergic cell culture. Eur J Pharmacol.

[CR65] Pickel VM, Joh TH, Field PM (1975). Cellular localization of tyrosine hydroxylase by immunohistochemistry. J Histochem Cytochem.

[CR66] Puschmann A (2017). New genes causing hereditary parkinson's disease or Parkinsonism. Curr Neurol Neurosci Rep.

[CR67] Radad K, Rausch WD, Gille G (2006). Rotenone induces cell death in primary dopaminergic culture by increasing ROS production and inhibiting mitochondrial respiration. Neurochem Int.

[CR68] Radad K, Moldzio R, Al-Shraim M (2015). Recent advances in autophagy-based neuroprotection. Expert Rev Neurother.

[CR69] Radad K, Al-Shraim M, Al-Emam A (2020). Autophagy inhibitor 3-methyladenine could not modulate rotenone neurotoxicity in primary mesencephalic cell culture. Int J Morphol.

[CR71] Rausch WD (1979) Enzymes of catechol- and indoleamine metabolism. Dissertation Technical University Vienna

[CR70] Rausch WD, Hirata Y, Nagatsu T (1988). Tyrosine hydroxylase activity in caudate nucleus from Parkinson's disease: effects of iron and phosphorylating agents. J Neurochem.

[CR72] Reichelt D, Radad K, Moldzio R (2016). Comparable neuroprotective effects of pergolide and pramipexole on ferrous sulfate-induced dopaminergic cell death in cell culture. CNS Neurol Disord Drug Targets.

[CR73] Ren X, Chen JF (2020). Caffeine and Parkinson’s disease: multiple benefits and emerging mechanisms. Front Neurosci.

[CR74] Riederer P, Rausch WD, Birkmayer W (1978). CNS Modulation of adrenal tyrosine hydroxylase in Parkinson's disease and metabolic encephalopathies. J Neural Transm Supplementum.

[CR75] Riederer P, Rausch WD, Schmidt B (1988). Biochemical fundamentals of Parkinson's disease. Mt Sinai J Med.

[CR76] Riederer P, Monoranu C, Strobel S (2021). Iron as the concert master in the pathogenic orchestra playing in sporadic Parkinson's disease. J Neural Transm.

[CR77] Salama M, Arias-Carrión O (2011). Natural toxins implicated in the development of Parkinson's disease. Ther Adv Neurol Disord.

[CR78] Salamon A, Dénes Z, László S (2020). Neuroprotection in Parkinson’s disease: facts and hopes. J Neural Trans.

[CR79] Scheller D, Stichel-Gunke C, Lübbert H (2008). Neuroprotective effects of rotigotine in the acute MPTP-lesioned mouse model of Parkinson's disease. Neurosci Lett.

[CR80] Shehadeh J, Double KL, Murphy KE (2019). Expression of tyrosine hydroxylase isoforms and phosphorylation at serine 40 in the human nigrostriatal system in Parkinson's disease. Neurobiol Dis.

[CR81] Sian-Hulsmann J, Monoranu C, Strobel S (2015). Lewy bodies: a spectator or salient killer?. CNS Neurol Disord Drug Targets.

[CR82] Sofic E, Riederer P, Heinsen H (1988). Increased iron (III) and total iron content in post mortem substantia nigra of parkinsonian brain. J Neural Transm.

[CR83] Swiątkiewicz M, Zaremba M, Joniec I (2013). Potential neuroprotective effect of ibuprofen, insights from the mice model of Parkinson's disease. Pharmacol Rep.

[CR84] Szökő É, Tábi T, Riederer P (2018). Pharmacological aspects of the neuroprotective effects of irreversible MAO-B inhibitors, selegiline and rasagiline, in Parkinson's disease. J Neural Transm.

[CR85] Takashima H, Tsujihata M, Kishikawa M (1999). Bromocriptine protects dopaminergic neurons from levodopa-induced toxicity by stimulating D_2_ receptors. Exp Neurol.

[CR86] Tolleson C, Claassen D (2012). The function of tyrosine hydroxylase in the normal and Parkinsonian brain. CNS Neurol Disord Drug Targets.

[CR87] Udagedara TB, Alahakoon AMBD, Goonaratna IK (2019). Vascular Parkinsonism: a review on management updates. Ann Indian Acad Neurol.

[CR88] Uddin R, Kim HH, Lee J-H (2013). Neuroprotective effects of medicinal plants. EXCLI J.

[CR89] Wakabayashi K, Tanji K, Mori F (2007). The Lewy body in Parkinson's disease: molecules implicated in the formation and degradation of alpha-synuclein aggregates. Neuropathology.

[CR90] Wakabayashi K, Tanji K, Odagiri S (2013). The Lewy body in Parkinson's disease and related neurodegenerative disorders. Mol Neurobiol.

[CR91] Wang J, Wang F, Mai D (2020). Molecular mechanisms of glutamate toxicity in Parkinson's disease. Front Neurosci.

[CR92] Yoshioka M, Tanaka K, Miyazaki I (2002). The dopamine agonist cabergoline provides neuroprotection by activation of the glutathione system and scavenging free radicals. Neurosci Res.

[CR93] Zhang SM, Hernan MA, Chen H (2002). Intakes of vitamins E and C, carotenoids, vitamin supplements, and PD risk. Neurology.

[CR94] Zhao X, Zhang M, Li C (2019). Benefits of Vitamins in the Treatment of Parkinson's Disease. Oxid Med Cell Longev.

[CR95] Zhou T, Zu G, Zhang X (2016). Neuroprotective effects of ginsenoside Rg1 through the Wnt/β-catenin signaling pathway in both in vivo and in vitro models of Parkinson's disease. Neuropharmacology.

[CR96] Zhu JL, Wu YY, Wu D (2019). SC79, a novel Akt activator, protects dopaminergic neuronal cells from MPP(+) and rotenone. Mol Cell Biochem.

